# The burden of stroke and its attributable risk factors in the Middle East and North Africa region, 1990–2019

**DOI:** 10.1038/s41598-022-06418-x

**Published:** 2022-02-17

**Authors:** Mehran Jaberinezhad, Mehdi Farhoudi, Seyed Aria Nejadghaderi, Mahasti Alizadeh, Mark J. M. Sullman, Kristin Carson-Chahhoud, Gary S. Collins, Saeid Safiri

**Affiliations:** 1 Social Determinants of Health Research Center, Department of Community Medicine, Faculty of Medicine, Tabriz University of Medical Sciences, Tabriz, Iran; 2 Student Research Committee, Tabriz University of Medical Sciences, Tabriz, Iran; 3 Neurosciences Research Center, Aging Research Institute, Tabriz University of Medical Sciences, Tabriz, Iran; 4 Department of Neurology, Faculty of Medicine, Tabriz University of Medical Sciences, Tabriz, Iran; 5 School of Medicine, Shahid Beheshti University of Medical Sciences, Tehran, Iran; 6 Systematic Review and Meta-Analysis Expert Group (SRMEG), Universal Scientific Education and Research Network (USERN), Tehran, Iran; 7 Department of Life and Health Sciences, University of Nicosia, Nicosia, Cyprus; 8 Department of Social Sciences, University of Nicosia, Nicosia, Cyprus; 9 Australian Centre for Precision Health, Allied Health and Human Performance, University of South Australia, Adelaide, SA Australia; 10 School of Medicine, The University of Adelaide, Adelaide, SA Australia; 11 Centre for Statistics in Medicine, NDORMS, Botnar Research Centre, University of Oxford, Oxford, UK; 12 NIHR Oxford Biomedical Research Centre, Oxford University Hospitals NHS Foundation Trust, Oxford, UK

**Keywords:** Stroke, Epidemiology

## Abstract

Stroke is one of the leading causes of mortality and morbidity across the globe. Providing comprehensive data on the burden of stroke in the Middle East and North Africa (MENA) could be useful for health policy makers in the region. Therefore, this article reported the burden of stroke and its attributable risk factors between 1990 and 2019 by age, sex, type of stroke, and socio-demographic index. Data on the point prevalence, death, and disability-adjusted life-years (DALYs), due to stroke, were retrieved from the Global Burden of Disease study 2019 for the 21 countries located in the MENA region from 1990 to 2019. The counts and age-standardised rates (per 100,000) were presented, along with their corresponding 95% uncertainty intervals (UIs). In 2019, the regional age-standardised point prevalence and death rates of stroke were 1537.5 (95% UI: 1421.9–1659.9) and 87.7 (78.2–97.6) per 100,000, which represent a 0.5% (− 2.3 to 1.1) and 27.8% (− 35.4 to − 16) decrease since 1990, respectively. Moreover, the regional age-standardised DALY rate in 2019 was 1826.2 (1635.3–2026.2) per 100,000, a 32.0% (− 39.1 to − 23.3) decrease since 1990. In 2019, Afghanistan [3498.2 (2508.8–4500.4)] and Lebanon [752.9 (593.3–935.9)] had the highest and lowest age-standardised DALY rates, respectively. Regionally, the total number of stroke cases were highest in the 60–64 age group and was more prevalent in women in all age groups. In addition, there was a general negative association between SDI and the burden of stoke from 1990 to 2019. Also, in 2019, high systolic blood pressure [53.5%], high body mass index [39.4%] and ambient particulate air pollution [27.1%] made the three largest contributions to the burden of stroke in the MENA region. The stroke burden has decreased in the MENA region over the last three decades, although there are large inter-country differences. Preventive programs should be implemented which focus on metabolic risk factors, especially among older females in low SDI countries.

## Introduction

Stroke is the second-leading cause of death (11.6% of total deaths) and the third-leading cause of death and disability combined (5.7% of total disability adjusted life years (DALYs))^1^. The economic cost of stroke is staggering, not only does recovery require complex and lengthy medical interventions and rehabilitation, but it also causes a significant loss of productivity^2^. The Organisation for Economic Co-operation and Development (OECD) estimates the average cost of medical care alone to range from 752 USD in Australia to 4850 USD in the United States^3^. As the epidemiologic transition happens in low- and middle-income countries, where the primary cause of morbidity and mortality changes from infectious diseases to non-communicable disease, there is expected to be an increase in the global burden of stroke, since at present there is no effective preventive measure which has been widely implemented^4,5^.

There is already growing concern as research has identified exceptionally high incidences of stroke, particularly among younger age cohorts, in middle-income countries such as Iran^6,7^. One population-based study (n = 624) of first-time stroke occurrences noted the age-specific stroke incidence were higher among young patients, than the rates observed in Western countries^6^. A systematic analysis of the Global Burden of Disease (GBD) 2019 study confirms these findings, with a 3.6 fold higher incidence of age-standardised stroke-related mortality in lower-income groups, compared to higher-income groups^8^. Moreover, the age-standardised DALY rate were 3.7 times higher for low-income groups, compared to high-income groups^8^. Meanwhile, evidence from the GBD 2016 project showed that although the global age-standardised death and point prevalence of stroke decreased from 1990 to 2016, the overall burden of stroke remained high^4^. With advancements in medical technology and the utilization of better diagnostic and therapeutic methods, the percentage of people surviving stroke has increased by as much as 84%. However, survivors often live with the long-term sequela, such as physical disabilities, neuropsychiatric problems, and speech and cognitive impairment^9–11^.

The global burden of stroke has been reported in previous studies, however this information is now out of date and the different subtypes of stroke have not previously been reported^4,12^. In addition, regional patterns may not follow the global patterns for stroke and regional policy makers need to focus on the region-specific patterns to avoid inappropriate decision making. Furthermore, as region-specific patterns may not be comprehensively described in papers reporting the global patterns^13^, there is a need for region-specific reports.

The Middle East and North Africa (MENA) region includes countries with wide differences in the level of social development. In addition, the rapidly changing social, economic, and cultural landscape of the MENA region further underlines the need for an updated and more detailed understanding of the burden of diseases. Although national-level studies have been conducted in some of the countries located in the MENA region, there is a dearth of research reporting the stroke burden at the regional level. One 2016 systematic review reported the epidemiology of stroke in the Middle East, however the data presented did not include all countries in the region and these dated results likely no longer reflect current trends, as the 64 included papers were published between 1980 and May 2015^14^. Therefore, the present study reported the point prevalence, death, and DALYs due to stroke and its attributable risk factors by age, sex, subtypes, and Socio-demographic Index (SDI; a composite of socio-demographic factors) in the MENA region from 1990 to 2019.

## Methods

GBD 2019, the most recent iteration of the project, estimated the burden of 369 diseases and 87 risk factors for 204 countries and territories, seven super-regions and 21 regions from 1990 to 2019. The general methodology of GBD 2019 and its improvements over previous iterations are described elsewhere^1^. Information on the fatal and non-fatal estimates can be obtained from https://vizhub.healthdata.org/gbd-compare/ and http://ghdx.healthdata.org/gbd-results-tool.

### Case definition and data inputs

Stroke was defined using the WHO criteria *rapidly developing clinical signs of focal (at times global) disturbance of cerebral function lasting more than 24 h or leading to death and of presumed vascular origin*^1,15^. However, data on transient ischemic attack (TIA) were not included^1^.

Stroke cases are considered acute from the day of the first stroke through to the 28th day after the event. Stroke cases are considered chronic 28 days after the stroke, while a chronic stroke includes the sequelae of an acute stroke and all recurrent stroke events. An episode of neurological dysfunction caused by focal cerebral, spinal, or retinal infarction was considered to be ischemic stroke. A focal collection of blood within the brain parenchyma or the ventricular system, which was not as a result of trauma, was considered to be an intracerebral hemorrhage. Finally, a subarachnoid hemorrhage consisted of bleeding into the subarachnoid space (the space between the arachnoid membrane and the pia mater of the brain or spinal cord)^1^. The International Classification of Diseases (ICD) 10 codes included in this research were: stroke (G45–G46.8, I60–I63.9, I65–I66.9, I67.0–I67.3, I67.5–I67.6, I68.1–I68.2, I69.0–I69.3), ischemic stroke (G45–G46.8, I63–I63.9, I65–I66.9, I67.2–I67.3, I67.5–I67.6, I69.3), intracerebral hemorrhage (I61–I62, I62.1–I62.9, I68.1–I68.2, I69.1–I69.2), and subarachnoid hemorrhage (I60–I60.9, I62.0, I67.0–I67.1, I69.0).

There was no systematic review performed for GBD 2019, as a systematic review was performed on the incidence, prevalence and mortality of stroke for GBD 2017. The search terms, dates, and databases used are shown in Table S1. Inpatient hospital data were included, which were adjusted for readmission and primary to any diagnosis using correction factors estimated from US claims data. Data were not included where the data points were implausibly low compared with other data from the same or similar locality. Moreover, unpublished stroke registry data for acute ischemic stroke, acute intracerebral hemorrhage, and acute subarachnoid hemorrhage were included. In addition, the study also included data from surveys. These surveys were identified using expert opinion and via a review of major survey series that were focused on world health and included a self-reported history of stroke. The list of data sources used for estimating ischemic stroke, intracerebral hemorrhage and subarachnoid hemorrhage have previously been reported^1^, and can be found here http://ghdx.healthdata.org/gbd-2019/data-input-sources.

Cerebrovascular disease (stroke) mortality was modelled using verbal autopsy and vital registration data. For those under 20 years of age, deaths were reassigned from verbal autopsy reports for cerebrovascular disease to the parent cardiovascular disease. The information regarding stroke type only used vital registration data, as accurate assessment of stroke type requires imaging studies, which are not commonly available in populations where the causes of death are determined by verbal autopsy. Data were excluded for any non-representative or subnational verbal autopsy data. In addition, data were also excluded where they were inconsistent with the rest of the data or created implausible time trends. Data from sources which were implausibly low in all age groups or caused the regional estimates to be improbably high were also excluded.

### Data processing and disease model

In GBD 2019, unspecified strokes (ICD-10 I64) were divided into ischemic stroke, intracerebral hemorrhage, and subarachnoid hemorrhage, based upon the proportions of each stroke in the original data. Furthermore, ICD-10 I62 was divided into intracerebral hemorrhage and subarachnoid hemorrhage, using the same method^1^. As with many GBD models, the diversity in the data sources included meant that adjustments were needed to the reference case definition. Therefore, the incidence and excess mortality data that differed from the reference case definitions were cross-walked using MR- BRT (a Bayesian meta-regression tool). Further detailed information on the MR-BRT have been presented previously^1^.

The Standard Cause of Death Ensemble model (CODEm) was used to estimate stroke mortality using data from vital registration and verbal autopsies. CODEm is a tool that models estimates of death for all locations across the time series (1990–2019) using geospatial relationships and information from covariates. The covariates used to model stroke mortality are presented in the appendix methods section of the GBD 2019 paper^1^. Following this, all available high quality data on the incidence, prevalence and mortality were fed into DisMod-MR 2.1, a Bayesian meta-regression tool, in order to model the incidence and prevalence of stroke across the region. DisMod-MR, a Bayesian geospatial disease modelling tool, which uses data on a number of disease parameters, uses the epidemiological relationships between these parameters, and geospatial relationships to estimate the prevalence and incidence of the disease or illness. Acute and chronic stroke were modelled separately for ischemic stroke, intracerebral hemorrhage, and subarachnoid hemorrhage, with further details on the modelling approach available elsewhere^1^.

### Compilation of results

The severity level, lay description, and stroke disability weights (DWs) for GBD 2019 are presented in Table S2. The years lived with disability (YLDs) was produced by multiplying the prevalence in each severity category with the severity-specific DWs. The years of life lost (YLLs) were then calculated by multiplying the number of deaths in an age group by the remaining life expectancy in that age group, as determined by the GBD standard life table. The YLLs and YLDs were then summed to calculate the DALYs. Uncertainty was propagated by sampling 1000 draws at each computational step and by combining uncertainty from multiple sources (i.e., input data, corrections of measurement error and estimates of residual non-sampling error). The uncertainty intervals (UIs) were defined as the 25th and 975th values of the ordered draws. The relationship between the burden of stroke, in terms of DALYs, and the SDI for the individual countries in the MENA region, were investigated using smoothing splines models^16^. SDI ranges from 0 (less developed) to 1 (most developed) and is comprised of the gross domestic product per capita (smoothed over the preceding 10 years), average years of schooling for the population older than 15 years of age, and the total fertility rate for those under 25 years old. R software (V 3.5.2) was used to map the age-standardised point prevalence, deaths and DALY rates.

### Risk factors

Those risk factors with evidence for stroke causation were included in the present study^17^. Furthermore, the percentage of DALYs were reported that were attributable to the following risk factors: high systolic blood pressure, high body mass index, high fasting plasma glucose, ambient particulate matter pollution, smoking, diet high in sodium, household air pollution, high low-density lipoprotein (LDL) cholesterol, kidney dysfunction, diet low in fruit, diet high in red meat, alcohol use, low temperature, lead exposure, diet low in fiber, secondhand smoke, diet low in vegetables, diet low in whole grains, low physical activity and high temperature^17^. The definitions of these risk factors and their relative risks have been previously reported^17^.

### Ethics approval

The present report was approved by ethics committee of Tabriz University of Medical Sciences (IR.TBZMED.REC.1400.453). This study is based on publicly available data and solely reflects the opinion of its authors and not that of the Institute for Health Metrics and Evaluation.

### Patient and public involvement

Patients and the public were not involved in the analyses or preparation of this manuscript.

## Results

### The Middle East and North Africa

In 2019, there were 7.3 million (95% UI: 6.8–7.9) prevalent cases of stroke in the MENA region, with an age-standardised point prevalence of 1537.5 (1421.9–1659.9) per 100,000 population, which represents a 0.5% decrease since 1990 (− 2.3 to 1.1) (Table 1; Table S3). Stroke accounted for 312.2 thousand (278.5–349.7) deaths in 2019, with an age-standardised rate of 87.7 (78.2–97.6) per 100,000 population, which is 27.8% lower than in 1990 (− 35.4 to − 16.0) (Table 1; Table S4). In 2019, the number of regional DALYs was 7.9 million (7.1–8.9), with an age-standardised rate of 1826.2 (1635.3–2026.2) DALYs per 100,000 population, a decrease of 32.0% since 1990 (− 39.1 to − 23.3) (Table 1; Table S5).

**Table 1:** Prevalent cases, deaths and DALYs due to stroke for both sexes in 2019 and percentage change of age-standardised rates during 1990–2019.

	Prevalence (95% UI)			Deaths (95% UI)			DALYs (95% UI)		
	counts (2019)	ASRs (2019)	Pcs in ASRs 1990–2019	Counts (2019)	ASRs (2019)	Pcs in ASRs 1990–2019	Counts (2019)	ASRs (2019)	Pcs in ASRs 1990–2019
Global	101,474,558 (93,211,910, 110,526,302)	1240.3 (1139.7, 1353)	− 6.1 (− 7.2, − 5)	6,552,725 (5,995,200, 7,015,139)	84.2 (76.8, 90.2)	− 36.4 (− 41.6, − 31.2)	143,232,184 (133,095,809, 153,241,824)	1768.1 (1640.6, 1889.4)	− 35.2 (− 40.5, − 30.5)
North Africa and Middle East	7,323,421 (6,794,727, 7,863,138)	1537.5 (1421.9, 1659.9)	− 0.5 (− 2.3, 1.1)	312,220 (278,450, 349,726)	87.7 (78.2, 97.6)	− 27.8 (− 35.4, − 16)	7,946,004 (7,060,209, 8,870,766)	1826.2 (1635.3, 2026.2)	− 32 (− 39.1, − 23.3)
Afghanistan	281,177 (260,881, 302,740)	1657.6 (1533.4, 1779.5)	3.8 (− 1.2, 8)	16,816 (11,747, 21,934)	161.5 (110.3, 208.5)	− 18.7 (− 36.1, − 1.6)	507,837 (375,947, 659,772)	3498.2 (2508.8, 4500.4)	− 21.8 (− 39.4, − 3.6)
Algeria	549,736 (506,627, 592,960)	1540.3 (1417.2, 1668)	− 10.7 (− 14.5, − 7)	24,811 (19,995, 30,219)	101.5 (82.6, 121.6)	− 43.4 (− 54.9, − 30.2)	543,945 (450,965, 649,230)	1755.4 (1459.6, 2081.9)	− 44.6 (− 55, − 31.9)
Bahrain	14,539 (13,369, 15,844)	1136.4 (1049, 1234.8)	− 24.2 (− 27.5, − 20.8)	245 (200, 310)	52.8 (43.5, 66.9)	− 48.5 (− 58.4, − 36.5)	7987 (6663, 9690)	940.9 (796.8, 1146.2)	− 52.5 (− 61.2, − 42.5)
Egypt	1,269,834 (1,170,811, 1,374,639)	1806.1 (1658.1, 1974.5)	18.5 (12.9, 23.9)	45,767 (33,557, 63,157)	85.7 (63.1, 118.9)	− 22.6 (− 42.6, 1.6)	1,493,970 (1,146,402, 1,946,512)	2138 (1636.8, 2809.8)	− 35.5 (− 50, − 17)
Iran (Islamic Republic of)	963,512 (859,232, 1,079,662)	1253.8 (1113.5, 1418.4)	− 13.3 (− 15.9, − 10.7)	40,912 (36,741, 43,849)	66.2 (58.7, 71.3)	− 45.1 (− 50.6, − 35.4)	884,768 (812,248, 943,655)	1262.2 (1153.5, 1346.3)	− 45.7 (− 51, − 38.3)
Iraq	520,023 (483,072, 557,268)	1968.8 (1823.4, 2122.1)	− 9.6 (− 13.3, − 5.4)	26,256 (21,422, 31,075)	143.3 (119.2, 166.1)	− 13.7 (− 30.2, 4.5)	682,943 (548,026, 826,273)	2922.9 (2399, 3459.1)	− 19.4 (− 35.5, − 0.8)
Jordan	134,580 (123,260, 145,467)	1793.9 (1621.2, 1952.9)	− 23.4 (− 27.7, − 19.5)	3367 (2758, 3983)	75.7 (61.3, 89)	− 49.8 (− 59.4, − 39.2)	86,118 (72,852, 100,150)	1448 (1220.4, 1679.5)	− 50.2 (− 58.7, − 41.1)
Kuwait	42,739 (39,334, 46,356)	1230.6 (1134.6, 1332.5)	− 5.9 (− 10.4, − 1.4)	920 (757, 1093)	46.5 (38, 55.2)	− 9.6 (− 24.2, 7.7)	25,463 (21,787, 29,824)	938.4 (795, 1093.3)	− 11.7 (− 24, 3.3)
Lebanon	74,841 (69,385, 80,771)	1425.1 (1320.3, 1538.1)	2.2 (− 1.8, 6.6)	1764 (1236, 2298)	35.2 (24.5, 45.6)	− 39.5 (− 55.5, − 19.2)	39,216 (30,874, 48,709)	752.9 (593.3, 935.9)	− 33.7 (− 47.6, − 16)
Libya	90,351 (83,440, 96,922)	1588.4 (1461.7, 1712.9)	14 (8.9, 18.7)	3086 (2364, 4016)	69.4 (53, 90.1)	− 17.9 (− 36.4, 7.9)	82,320 (64,181, 104,686)	1570.5 (1237.3, 2001.2)	− 21.3 (− 37.5, 1.4)
Morocco	557,119 (515,485, 599,727)	1695.1 (1563.4, 1833.2)	3.4 (− 0.7, 8.2)	29,033 (23,331, 35,135)	116.4 (94.2, 139.3)	− 11.9 (− 29.1, 8)	666,284 (534,574, 808,810)	2257.8 (1838.8, 2702.5)	− 17.2 (− 33.9, 0.9)
Oman	37,722 (34,606, 41,220)	1525.6 (1399.9, 1686.6)	− 8.5 (− 12.4, − 4.5)	1030 (895, 1196)	103.7 (90.7, 118.7)	− 28.8 (− 45, − 3.7)	30,859 (27,010, 36,651)	1884.3 (1667.3, 2127.1)	− 38.7 (− 51.9, − 20.7)
Palestine	41,305 (37,846, 45,067)	1510.8 (1381.5, 1666.5)	− 3.7 (− 8.5, 1.4)	2019 (1740, 2286)	122.4 (105.6, 138.2)	− 29.1 (− 43.5, − 10.1)	44,902 (39,457, 50,763)	2128.3 (1878.2, 2399.7)	− 32.5 (− 46.4, − 15)
Qatar	22,335 (20,296, 24,525)	1226.3 (1127.4, 1327.2)	− 22.2 (− 25.6, − 18.9)	181 (136, 239)	52.9 (42.5, 69.2)	− 36.7 (− 54, − 15.7)	8998 (7254, 11,097)	904.5 (747.6, 1130.3)	− 44.7 (− 57.5, − 29.5)
Saudi Arabia	480,500 (442,193, 523,176)	1967.7 (1818.1, 2143.5)	7.9 (− 0.1, 15.2)	12,669 (9750, 15,359)	102.7 (80.4, 120.5)	− 32.5 (− 49.1, − 10)	417,599 (326,188, 509,857)	2114.9 (1685.1, 2485.5)	− 29.1 (− 46.4, − 5.6)
Sudan	398,038 (367,084, 427,930)	1785.8 (1645.2, 1936.9)	8.2 (3.7, 12.8)	19,638 (14,425, 27,765)	125.2 (92.6, 174.9)	− 29.4 (− 42.2, − 12)	522,412 (385,879, 715,571)	2585.5 (1970.5, 3552.9)	− 33 (− 45.3, − 16.9)
Syrian Arab Republic	199,599 (184,381, 215,112)	1518.7 (1403.8, 1637.5)	− 13.9 (− 17.3, − 10.5)	9186 (7117, 11,838)	99 (78.3, 124.9)	− 32.2 (− 50.2, − 8.6)	238,333 (184,920, 304,410)	2018.1 (1589.6, 2547.1)	− 40.2 (− 55.3, − 19.9)
Tunisia	159,928 (147,442, 173,396)	1260.8 (1162.6, 1366)	22.1 (16.3, 28.2)	8713 (6627, 11,147)	80 (60.6, 101.4)	− 25 (− 43.8, − 0.6)	176,891 (135,395, 225,499)	1477.5 (1138.5, 1875)	− 24.7 (− 42.8, − 2)
Turkey	1,080,379 (1,002,616, 1,164,932)	1213.6 (1124.7, 1309.4)	− 8.7 (− 12.4, − 4.6)	48,947 (39,204, 59,511)	60.6 (48.7, 73.6)	− 16.5 (− 39.5, 6.2)	993,082 (820,881, 1,177,528)	1162.6 (965, 1380.4)	− 23.5 (− 42.5, − 5.2)
United Arab Emirates	136,808 (125,534, 148,340)	2225.7 (2037.3, 2436.3)	− 7.5 (− 11.6, − 3.6)	2168 (1544, 3026)	91.3 (70.2, 118.7)	− 50 (− 62, − 34.9)	95,518 (70,890, 128,945)	1925.6 (1517, 2457.9)	− 46 (− 58.5, − 30.5)
Yemen	260,915 (242,029, 280,821)	1609.4 (1480.2, 1745.5)	0.8 (− 4.2, 5.7)	14,375 (10,909, 18,865)	135.7 (102.4, 176.6)	− 22.1 (− 38.2, 0.7)	388,486 (302,567, 500,074)	2765.5 (2161.6, 3520.5)	− 24.5 (− 41, − 1.9)

### National level

**Figure 1:**
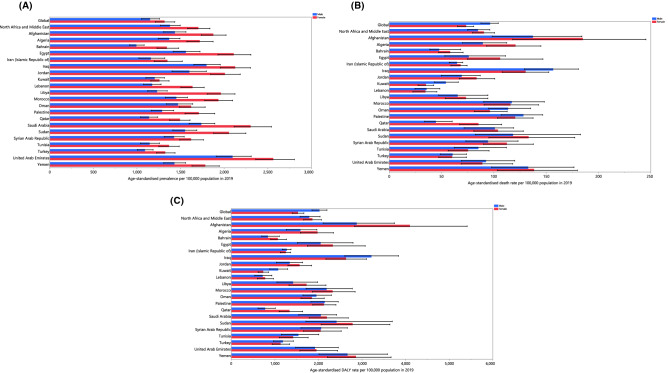
Age-standardised point prevalence (**A**), death (**B**), and DALYs (**C**) of stroke (per 100,000 population) in the Middle East and North Africa region in 2019, by sex and country. DALY= disability-adjusted-life-years. (Generated from data available from http://ghdx.healthdata.org/gbd-results-tool).

In 2019, the national age-standardised point prevalence of stroke among the countries that comprise the MENA region ranged from 1136.4 to 2225.7 cases per 100,000 population. The United Arab Emirates (UAE) [2225.7 (2037.3–2436.3), Iraq [1968.8 (1823.4–2122.1)] and Saudi Arabia [1967.7 (1818.1–2143.5)] had the three highest age-standardised point prevalences of stroke in 2019. In contrast, Bahrain [1136.4 (1049–1234.8)], Turkey [1213.6 (1124.7–1309.4)] and Qatar [1226.3 (1127.4–1327.2)] had the lowest rates (Fig. 1A; Table S3).

The national age-standardised death rates of stroke in 2019 varied from 35.2 to 161.5 cases per 100,000 population in the countries located in the MENA region. The highest rates were observed in Afghanistan [161.5 (110.3 to 208.5)], Iraq [143.3 (95% UI: 119.2 to 166.1)] and Yemen [135.7 (102.4–176.6)], while the lowest rates were found in Lebanon [35.2 (24.5–45.6)], Kuwait [46.5 (38–55.2)] and Bahrain [52.8 (43.5–66.9)] (Fig. 1B; Table S4).

In 2019, the national age-standardised DALY rate of stroke among the MENA countries ranged from 752.9 to 3498.2 cases per 100,000 population. The highest rates were observed in Afghanistan [3498.2 (2508.8–4500.4)], Iraq [2922.9 (2399–3459.1)] and Yemen [2765.5 (2161.6–3520.5)]. Conversely, the lowest rates were seen in Lebanon [752.9 (593.3–935.9)], Saudi Arabia [904.5 (747.6–1130.3)] and Kuwait [938.4 (795–1093.3)] (Fig. 1C; Table S5).

The percentage change in the age-standardised point prevalence, from 1990 to 2019, showed a decrease in some of the MENA countries, with Bahrain [− 24.2 (− 27.5 to − 20.8)], Jordan [− 23.4 (− 27.7 to − 19.5)] and Qatar [− 22.2 (− 25.6 to − 18.9)] having the highest decreases and Tunisia [22.1 (16.3–28.2)], Egypt [18.5 (12.9–23.9)] and Libya [14.0 (8.9–18.7)] having the highest increases (Table S3; Fig. S1).

Additionally, no country was observed to have an increase in the age-standardised death or DALY rates of stroke from 1990 to 2019. The UAE [− 50.0 (− 62.0 to − 34.9)], Jordan [− 49.8 (− 59.4 to − 39.2)] and Bahrain [− 48.5 (− 58.4 to − 36.5)] showed the largest decreases in the age-standardised death rates of stroke over the measurement period (Table S4; Fig. S2). In contrast, Bahrain [− 52.5 (− 61.2 to − 42.5)], Jordan [− 50.2 (− 58.7 to − 41.1)] and the UAE [− 46.0 (− 58.5 to − 30.5)] showed the largest decreases in the age-standardised DALY rates over the same period (Table S5; Fig. S3).

### Age and sex patterns

**Figure 2:**
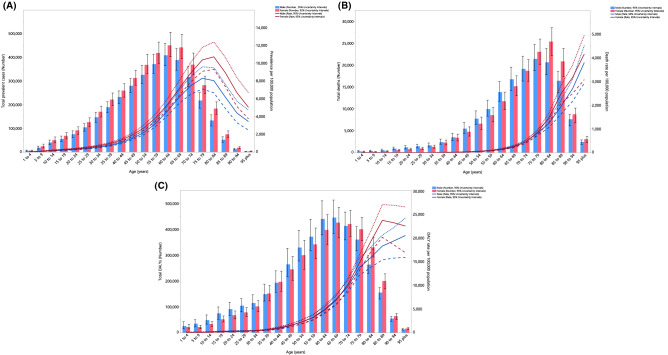
Numbers of prevalent cases and prevalence (**A**), number of deaths and death rate (**B**) and the number of DALYs and DALY rate (**C**) for stroke per 100,000 population in the Middle East and North Africa region, by age and sex in 2019; Dotted and dashed lines indicate 95% upper and lower uncertainty intervals, respectively. DALY= disability-adjusted-life-years. (Generated from data available from http://ghdx.healthdata.org/gbd-results-tool).

In 2019, the regional point prevalence of stroke was highest in the 75–79 and 80–84 age groups in females and males, respectively. Similarly, the number of prevalent cases was highest in the 60–64 age group. Furthermore, the global point prevalence and number of prevalent cases of stroke were higher in females of all ages (Fig. 2A). In 2019, the regional death rate of stroke was highest in the oldest age group. The number of deaths reached its highest in the 75–79 and 80–84 age groups for males and females, respectively (Fig. 2B). There was a clear increase in the regional DALY rate of stroke for females, up to the 85–89 age group, followed by a decrease up to the oldest age group (95^+^). In contrast, the regional DALY rate for males increased with advancing age up to the 95^+^ age group. In addition, the number of DALYs was highest in the 65–69 age group, for both males and females (Fig. 2C).

In 2019, ischemic stroke and subarachnoid hemorrhage were the most and least common types of stroke, in terms of prevalence, deaths and DALYs (Figs. S4, S5, S6). The highest prevalent cases and point prevalence (per 100,000) of ischemic stroke were found in the 65–69 and 80–84 age groups, respectively. The highest point prevalence of intracerebral hemorrhage and subarachnoid hemorrhage (per 100,000) were found in the 65–69 and 50–54 age groups, respectively (Fig. S4). The age-standardised death and DALY rates of all three types of stroke increased with advancing age (up to 95^+^), but the largest increase was for ischemic stroke, followed by intracerebral hemorrhage and subarachnoid hemorrhage (Figs. S5, S6).

**Figure 3:**
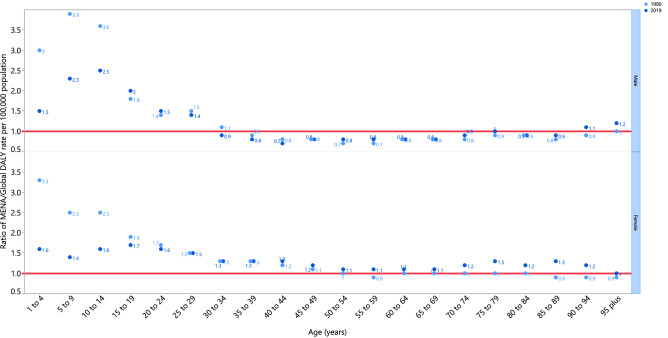
Ratio of the Middle East and North Africa region to the global stroke DALY rate according to age group and sex, 1990–2019. DALY= disability-adjusted-life-years. (Generated from data available from http://ghdx.healthdata.org/gbd-results-tool).

The rate ratio, comparing the age-standardised DALY rates in MENA to the global rates for the different age groups by sex in 1990 and 2019, showed that there were substantial variations from the reference values in males and females less than 30 years old, while all other age groups were similar to the global average. In other words, those younger than 30 years old in the MENA region had a higher burden of stroke than the global average, for both 1990 and 2019. Moreover, in 2019 males aged 30–74 years old had a lower burden of stroke than the global average, while females in all age groups had an equal or higher burden of stroke, compared with the global average (Fig. 3).

### Association with the socio-demographic index (SDI)

**Figure 4:**
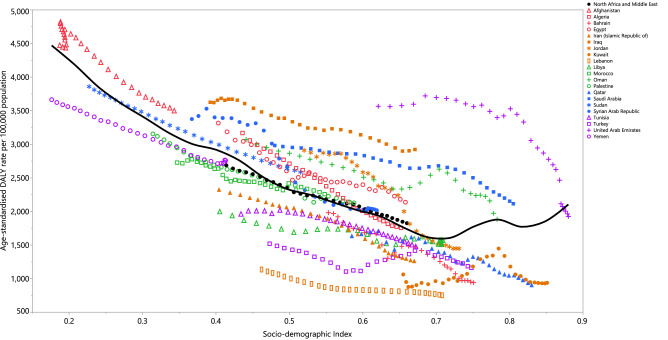
Age-standardised DALY rates of stroke for 21 countries and territories, by SDI in 2019; Expected values based on the Socio-demographic Index and disease rates in all locations are shown as the black line. Each point shows the observed age-standardised DALY rate for each country in 2019. DALY= disability-adjusted-life-years. SDI= Socio-demographic Index (Generated from data available from http://ghdx.healthdata.org/gbd-results-tool).

From 1990 to 2019, the burden of stroke generally decreased with increasing socio-economic development. Countries, such as Afghanistan, Egypt, Iraq, Jordan and Saudi Arabia had much higher than expected burdens, whereas countries such as Lebanon, Kuwait, Qatar, Bahrain and Turkey had much lower than expected burdens (Fig. 4).

### Risk factors

**Figure 5:**
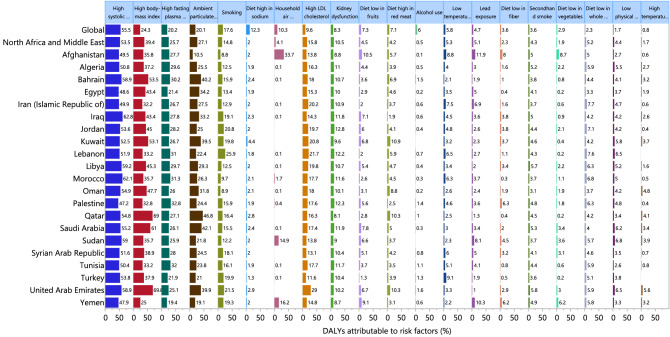
Percentage of DALYs due to stroke attributable to risk factors for the Middle East and North Africa countries in 2019. DALY=disability adjusted life years (Generated from data available from http://ghdx.healthdata.org/gbd-results-tool).

The proportion of DALYs due to stroke, which were attributable to the individual risk factors, differed across the MENA countries. At the regional level high systolic blood pressure [53.5%], high body mass index (BMI) [39.4%] and ambient particulate air pollution [27.1%] were the main contributors to the stroke burden (Fig. 5). For males, high systolic blood pressure [52.4%], high body mass index [37.7%] and ambient particulate air pollution [28.0%] had the highest contributions to the DALYs due to stroke (Fig. S7). The three main contributors for females were high systolic blood pressure [54.6%], high body mass index [41.2%] and high fasting plasma glucose [26.5%] (Fig. S8).

## Discussion

The present study showed that the age-standardised point prevalence of stroke in the region did not substantially change from 1990 to 2019. However, the age-standardised death rate decreased dramatically, from 121.4 in 1990 to 87.7 in 2019 (i.e. − 27.8%). An even larger decrease was observed in the associated age-standardised DALY rate, which decreased from 2685.3 in 1990 to 1826.2 in 2019 per 100,000 population (i.e. − 32.0%). Overall, the burden of stroke in the MENA region had a positive association with increasing age and a negative association with SDI level. Moreover, the DALYs attributable to high BMI, high fasting plasma glucose and ambient particulate air pollution were higher in the region than the global average.

In accordance with our findings, the GBD 2019 Stroke paper reported that despite a 32.0% global increase in the total DALYs over the period 1990–2019, the age-standardised rate per 100,000 decreased by 36%^13^. In comparison to the 2016 results, we found lower DALYs, after accounting for the lower mortality rates, which may be due to better post-stroke care than three years ago, at least in some countries.

At the national level, Kuwait had higher DALYs for males, which may be due to population aging, as the female population has not aged to the point of being at an increased risk of vascular events, such as stroke. Compared to other countries, Afghanistan, Yemen and Iraq had higher mortality rates, which could be as a result of ongoing conflicts and lower access to both preventive measures and acute stroke care^18^. The important objective for these countries should be the restoration of health systems after conflicts, since many efforts tend to fail while the war continues^19,20^.

There is a substantial gap between the global and the regional stroke burden for those in the lower ages (i.e. 1–29 age groups), suggesting that there are factors driving up the stroke rates among younger adults in the MENA region. Previous studies have shown that the most important risk factors in young adults are low physical activity and hypertension, so new criteria might be required to assess the risk factors in younger patients^21–23^. As the prevalence of comorbidities and stroke risk factors, such as hypertension, hyperlipidemia, diabetes and cardiac diseases increase with population aging, stroke is expected to have a higher burden in the elderly^24^, as found in our report.

A holistic view is required to analyse the prevalence, burden, and death rate changes over the years in each individual country, as each country has adopted its own preventive methods and there are different social determinants affecting the population in each country. A noteworthy point to consider is the heterogeneity of population distributions in the countries that comprise the MENA region. Countries, like Afghanistan, are predominantly agriculture-based societies which have a substantial proportion of the population living in rural areas, while others (e.g., UAE) have transformed into industrialised societies. These factors can both profoundly affect lifestyle and access to treatment.

The present study found lower DALY rates for countries with higher SDI levels. However, the increase observed in the DALYs by SDI figure, from an SDI of about 0.7, could suggest that higher SDI rates may result in more sedentary lifestyles and higher BMIs in populations with higher income and higher socioeconomic status^25^. However, the increase seen above 0.7 may simply be due to the quality of the data, since only one country in the MENA region has an SDI > 8 (i.e. the UAE). Furthermore, the UAE had high DALYs across the reporting period, and the DALYs decreased with increases in the UAE’s SDI. In this regard, the study by Avan et al. reported that the burden of stroke has decreased in regions with different socioeconomic statuses, but the reduction was more rapid in countries with higher socioeconomic status^26^.

High systolic blood pressure remains the number one risk factor for stroke in both the male and female populations and therefore must be the first priority for primary prevention efforts. This is followed by high BMI and ambient particulate air pollution. The analysis of the risk factors was again in line with global results, although particulate matter was the third most important risk factor, instead of the second^13^. This pattern differed in Qatar, Saudi Arabia and the UAE, where high BMI was the most important risk factor. The results of longitudinal studies show that physical activity, healthy diet, smoking cessation and the management of hypertension can lead to a reduction in the risk of stroke^27^. In the Eastern Mediterranean Region (EMR), Qatar, which has 78.1% of the population classified as overweight, was also the leading country in this region in 2014^28^. Furthermore, our research found that high BMI contributed 69.0% to the DALYs due to stroke in Qatar. Moreover, the prevalence of smoking in the MENA region was higher than the global average, with an age-standardised prevalence rate of 5.63 and 32.40 per 100,000 population in females and males, respectively^29^. Therefore, it should not be surprising that our results showed that 17.6% of stroke DALYs were attributable to smoking.

Health system efforts can be divided into several categories; primordial, primary, secondary and tertiary prevention, which includes acute care, chronic care, and rehabilitation^30^. However, before any planning for expanding or reforming the delivery of such services takes place, it is vital to consider the cultural context, as most of the populations in the region are considered traditional societies. Notably, the universal health coverage to access healthcare services and improving healthcare delivery in terms of comprehensiveness, quality and perceptions should be also taken into consideration.

With very little regard to the social determinants of health in the MENA region, primordial prevention is only limited to what the healthcare system can provide, in contrast to the multi-sectoral efforts required to change people’s health-related behavior, air pollution, living conditions, access to quality food and water, etc^31^. Another point of concern for the whole region is the sand storms and subsequent increase in particulate matter. Countries must be prepared to both address this problem and to mitigate its effects^32^.

A 2021 report by the World Health Organization identified urgent need to improve access to stroke units and services globally, particularly among low- and middle-income countries^33^. Following the evaluation, which compared data on stroke services across 84 countries, it was identified that fewer activities, such as registries, recent risk factor surveys and participation in research were observed in low-income countries, compared to high-income countries. Acute stroke treatments were observed in around 60% of high-income countries, compared to 26% of low-income countries. The recommendation was that a framework be established that supports regular monitoring of the stroke burden and services, implementation of prevention activities and essential acute stroke care services and the provision of interdisciplinary care for stroke rehabilitation^33^.

Primary prevention is usually the approach most valued in the health systems and the most obvious targets for this are hypertension, diabetes mellitus, high BMI, and dyslipidemia^24,34^. However, active patient screening and education on preventive measures are often performed infrequently and are perhaps only associated with specific campaigns, as most physicians rarely have enough time to carefully assess each patient’s individual situation and tailor-make a strategy for them. Moreover, the quality of guidelines for stroke management in low- and middle-income countries have been evaluated in a systematic review, which found that none recommended surveillance and few covered primary stroke prevention^35^. Indeed, many of the 108 eligible guidelines from across 47 countries fell short on the required standards for guideline development, breadth of target audience, coverage of the four components of stroke services and adaptation to socioeconomic context; with less than a quarter detailing implementation plans and socioeconomic considerations^35^.

Better communication methods are also required to encourage the population to take more responsibility for their own health. In the high-income country of New Zealand, participants from a 2020 study inaccurately reported that stroke was not a major cause of death (identified by only 1.5% of participants)^36^. Awareness of stroke risk factors was also low, particularly among individuals with lower education and income. The authors recommend that culturally tailored awareness campaigns may help to improve knowledge of stroke risk factors and help community members recognise stroke as being a major cause of death^36^. Obviously, no preventive measure is meaningful without voluntary involvement from the population. However, unless efforts are made to mitigate risk factors through primary stroke and cardiovascular prevention strategies, the global burden of stroke will continue to rise^37^. The plethora of barriers aside, there is no shortage of effective primary prevention interventions that have proven to be effective, as shown in a recent Cochrane review^38^.

As for secondary prevention, educational and behavioral interventions have been thought to further lower stroke DALYs, by improving factors such as patient adherence to medications. However, evidence indicates that these interventions do not result in clear improvements in controlling risk factors, such as blood pressure^39^. The current focus of studies has been mainly on post stroke treatment options, such as different anti platelet and anticoagulants, antihypertension medications, control of dyslipidemia and treating definitive underlying causes, such as patent foramen ovale. However, all of the aforementioned focus areas are limited by their availability and affordability^40^.

Treatment has improved drastically over the years, especially with the widespread use of recombinant tissue plasminogen activator (rTPA), which has substantially reduced mortality^41^. However, a meta-analysis of individual-patient data showed that intravenous thrombolysis with alteplase was not significantly associated with a reduction in the 90-day mortality rate, compared with the control group (17.9% vs. 16.5%; hazard ratio (HR): 1.11; 95% CI 0.99–1.25)^42^. Nowadays, many efforts are made to reduce the time gap between the onset of symptoms and the administration of the appropriate medication, particularly the door to needle time^43^. However, there is substantial disparity in the access to quality acute care facilities, with poor availability in low income countries. As the ICD11 includes imaging criteria for stroke and new studies show the improved outcomes associated with complementary imaging techniques, such as perfusion imaging, hospitals will have to plan for the expansion of their care packages for stroke patients in the future^44^. New home-based methods, such as telerehabilitation, robotic devices, and virtual reality devices have added a new frontier to rehabilitation efforts and appear promising leads to follow in the future^45^. However, evidence supporting these approaches are producing mixed results, with benefits found to be dose-dependent and therefore ongoing compliance is essential^46^. Self-management can also be considered among the interventions to improve rehabilitative services, as this has been shown to improve outcomes^47^.

One of the main focuses of the present study was to report the data for the different stroke subtypes. As expected, ischemic stroke was the most prevalent type, followed by hemorrhagic stroke and subarachnoid hemorrhage, respectively. Our results are in line with global results and previous observations^13^.

As with all studies, this research has a number of limitations. The most pertinent limitation is a lack of high-quality primary data, which means that the results are mostly dependent on the modeling and estimation methods applied. The implementation of better registry systems with standard criteria would greatly help to alleviate this problem. It should again be noted that the results should be interpreted with some caution, given the paucity of high quality population based studies for many of the countries included in the present study. This is particularly a problem in the low-income countries, and those afflicted by political and therefore health service upheaval. Moreover, the data on a sub-national level, based on the area of residence (i.e. rural or urban areas), are not yet available. These limitations should be taken into account in further GBD iterations.

## Conclusions

The burden of stroke in MENA has decreased over the last 30 years, in line with the global trend, but with a less steep decline. Although there were inter-country variations in the epidemiology of stroke in the MENA countries, most of the countries showed a decline. The present findings suggest that regional policy makers should prioritize older adults and females for preventative programs, especially in countries with lower socio-economic levels. Moreover, preventative and therapeutic programs should aim to address metabolic risk factors, like high systolic blood pressure and high BMI. Future studies are needed which estimate the prevalence of stroke over the next few decades, to enable planning for future resource allocation and stroke management.

## Supplementary Information

Supplementary Figure S1.

Supplementary Figure S2.

Supplementary Figure S3.

Supplementary Figure S4.

Supplementary Figure S5.

Supplementary Figure S6.

Supplementary Figure S7.

Supplementary Figure S8.

Supplementary Table S1.

Supplementary Table S2.

Supplementary Table S3.

Supplementary Table S4.

Supplementary Table S5.

## Data Availability

The data used for these analyses are all publicly available.
