# Supplementary material for: The burden of stroke and its attributable risk factors in the Middle East and North Africa region, 1990–2019
Source: Sci Rep. 2022 Feb 17;12:2700. doi: 10.1038/s41598-022-06418-x (PMC8854638; doi:10.1038/s41598-022-06418-x)
Supplement: Supplementary file 4 — Supplementary Figure S4. [file 41598_2022_6418_MOESM4_ESM.pdf]

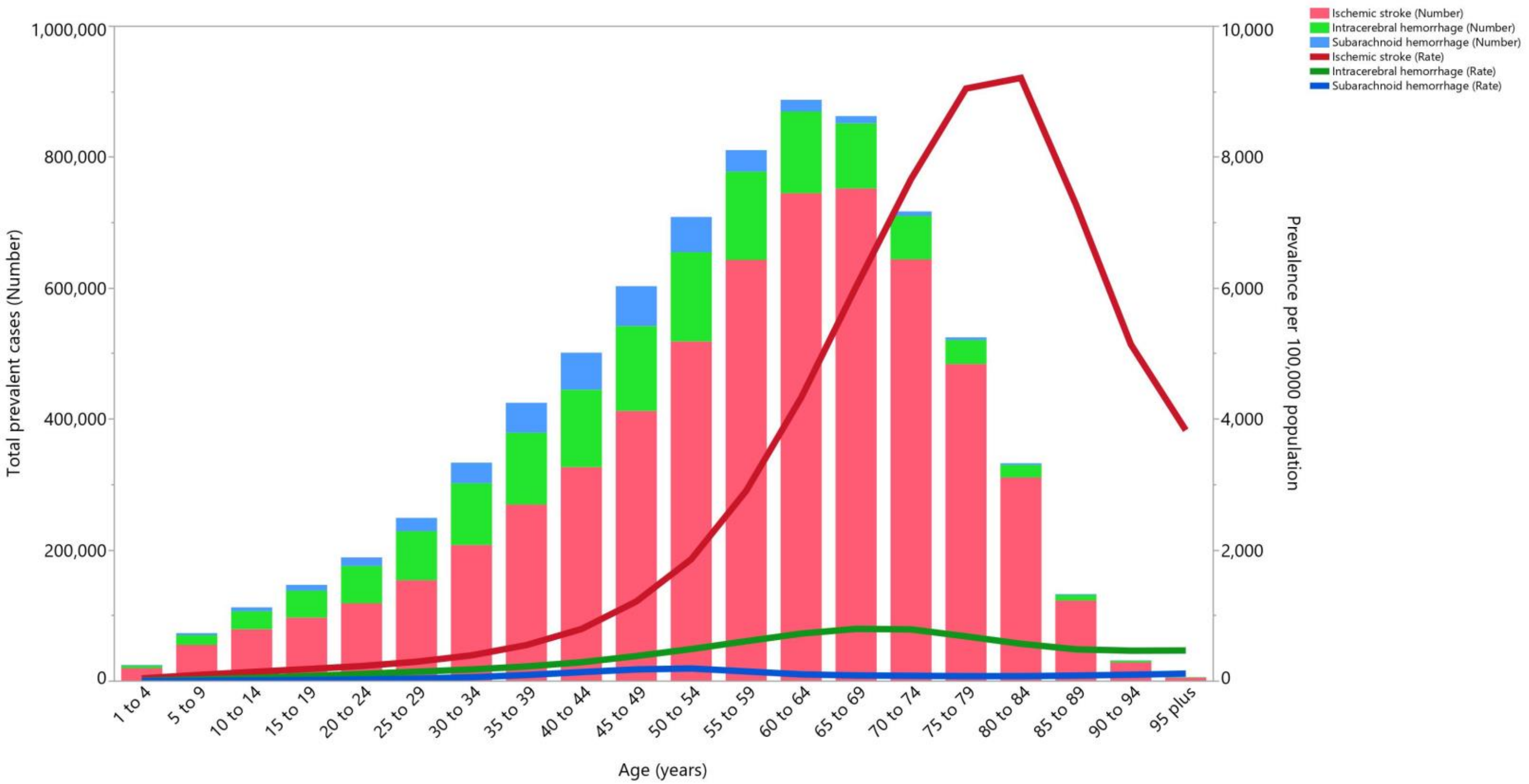

Figure S4: Numbers of prevalent cases and prevalence for stroke per 100,000 population in the Middle East and North Africa region, by age and type in 2019; Dotted and dashed lines indicate 95% upper and lower uncertainty intervals, respectively. (Generated from data available from http://ghdx.healthdata.org/gbd-results-tool).
