# Supplementary material for: The burden of stroke and its attributable risk factors in the Middle East and North Africa region, 1990–2019
Source: Sci Rep. 2022 Feb 17;12:2700. doi: 10.1038/s41598-022-06418-x (PMC8854638; doi:10.1038/s41598-022-06418-x)
Supplement: Supplementary file 7 — Supplementary Figure S7. [file 41598_2022_6418_MOESM7_ESM.pdf]

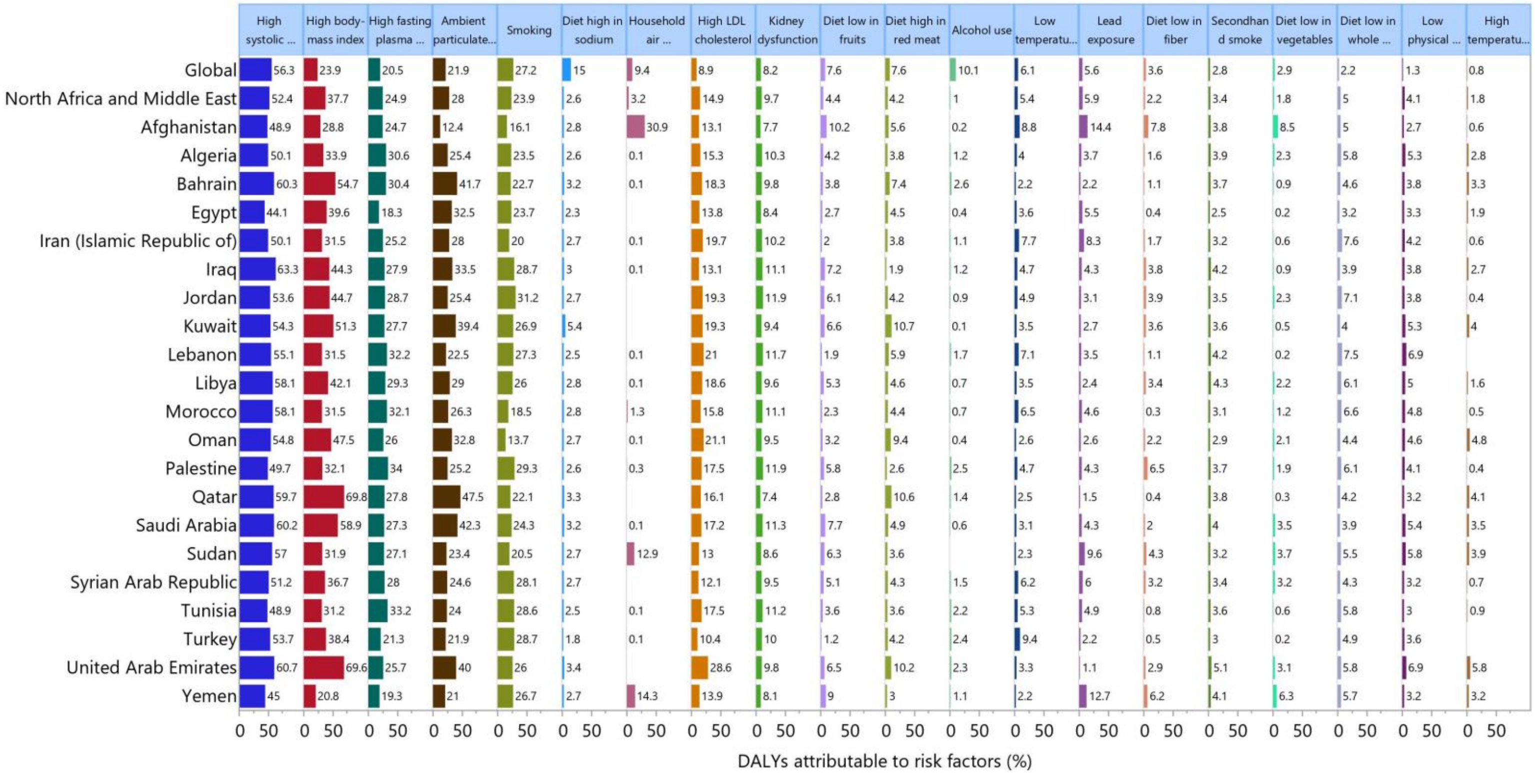

Figure S7: Percentage of DALYs due to stroke attributable to risk factors for the Middle East and North Africa countries, males, in 2019.

DALY=disability adjusted life years (Generated from data available from http://ghdx.healthdata.org/gbd-results-tool).
