# Supplementary material for: The burden of stroke and its attributable risk factors in the Middle East and North Africa region, 1990–2019
Source: Sci Rep. 2022 Feb 17;12:2700. doi: 10.1038/s41598-022-06418-x (PMC8854638; doi:10.1038/s41598-022-06418-x)
Supplement: Supplementary file 9 — Supplementary Table S1. [file 41598_2022_6418_MOESM9_ESM.pdf]

Table S1: Search terms, dates of search, and databases queried to estimate the burden of stroke
**Ischemic stroke**<br><br>a. Google scholar: ("ischemic stroke" OR “cerebral infarction” OR “ischaemic stroke”) AND (incidence OR prevalence OR mortality OR epidemiology). Reviewed first 1000 hits, sorted by relevance<br><br>b. Global Index Medicus search: (tw:("ischemic stroke") OR tw:(“cerebral infarction” OR tw:(“ischaemic stroke”)) AND (tw:(incidence) OR tw:(prevalence) OR tw:(mortality) OR tw:(epidemiology)) AND NOT (tw:(rats) OR tw:(mice) OR tw:(dogs) OR tw:(apes) OR tw:(monkeys)). Dates of search: 01 Jan 2010 – 31 Aug 2017
**Intracerebral hemorrhage**<br><br>a. Google scholar: ("hemorrhagic stroke" OR “intracerebral hemorrhage” OR “haemorrhagic stroke” OR “intracerebral haemorrhage”) AND (incidence OR prevalence OR mortality OR epidemiology). Reviewed first 1000 hits, sorted by relevance<br><br>b. GIM search: (tw:("intracerebral hemorrhage") OR tw:(“intracerebral haemorrhage”) OR tw:(“hemorrhagic stroke”) OR tw:(“haemorrhagic stroke”)) AND (tw:(incidence) OR tw:(prevalence) OR tw:(mortality) OR tw:(epidemiology)) AND NOT (tw:(rats) OR tw:(mice) OR tw:(dogs) OR tw:(apes) OR tw:(monkeys)). Dates of search: 01 Jan 2010 –31 Aug 2017
**Subarachnoid hemorrhage**<br><br>a. Google scholar search: ("subarachnoid hemorrhage" OR “subarachnoid haemorrhage”) AND (incidence OR prevalence OR mortality OR epidemiology). Reviewed first 1000 hits, sorted by relevance.<br><br>b. GIM search: (tw:("subarachnoid hemorrhage") OR tw:(“subarachnoid haemorrhage”)) AND (tw:(incidence) OR tw:(prevalence) OR tw:(mortality) OR tw:(epidemiology)) AND NOT (tw:(rats) OR tw:(mice) OR tw:(dogs) OR tw:(apes) OR tw:(monkeys)). Dates of search: 01 Jan 2010 – 31 Aug 2017
