# Supplementary material for: The burden of stroke and its attributable risk factors in the Middle East and North Africa region, 1990–2019
Source: Sci Rep. 2022 Feb 17;12:2700. doi: 10.1038/s41598-022-06418-x (PMC8854638; doi:10.1038/s41598-022-06418-x)
Supplement: Supplementary file 10 — Supplementary Table S2. [file 41598_2022_6418_MOESM10_ESM.pdf]

| Table S2: Severity level, lay description, and disability weights for stroke in GBD 2019 | | | | |
|---|---|---|---|---|
| **Acute Stroke** | | | | |
| **Severity level** | **Lay description** | **Modified Rankin scale** | **Cognitive status** | **Disability weight (95% CI)** |
| **Mild** | Has some difficulty in moving around and some weakness in one hand, but is able to walk without help. | 1 | N/A | 0.019 (0.01–0.032) |
| **Moderate** | Has some difficulty in moving around, and in using their hands for lifting and holding things, dressing, and grooming. | 2,3 | MoCA>=24 or MMSE>=26 | 0.07 (0.046–0.099) |
| **Moderate plus cognition problems** | Has some difficulty in moving around, in using their hands for lifting and holding things, dressing and grooming, and in speaking. The person is often forgetful and confused. | 2,3 | MoCA<24 or MMSE<26 | 0.316 (0.206–0.437) |
| **Severe** | Is confined to bed or a wheelchair, has difficulty speaking, and depends on others for feeding, toileting, and dressing. | 4, 5 | MoCA>=24 or MMSE>=26 | 0.552 (0.377–0.707) |
| **Severe plus cognition problems** | Is confined to bed or a wheelchair, depends on others for feeding, toileting, and dressing, and has difficulty speaking, thinking clearly, and remembering things. | 5? | MoCA<24 or MMSE<26 | 0.588 (0.411–0.744) |
| **Chronic Stroke** | | | | |
| **Severity level** | **Lay description** | **Modified Rankin scale** | **Cognitive status** | **Disability weight (95% CI)** |
| **Asymptomatic** | | 0 | N/A | N/A |
| **Long-term consequences, mild** | Has some difficulty in moving around and some weakness in one hand, but is able to walk without help. | 1 | N/A | 0.019 (0.01–0.032) |
| **Long-term consequences, moderate** | Has some difficulty in moving around, and in using their hands for lifting and holding things, dressing, and grooming. | 2, 3 | MoCA>=24 or MMSE>=26 | 0.07 (0.046–0.099) |
| **Long-term consequences, moderate plus cognition problems** | Has some difficulty in moving around, in using their hands for lifting and holding things, dressing and grooming, and in speaking. The | 2, 3 | MoCA<24 or MMSE<26 | 0.316 (0.206–0.437) |

| | | | | |
|---|---|---|---|---|
| | person is often forgetful and confused. | | | |
| **Long-term consequences, severe** | Is confined to bed or a wheelchair, has difficulty speaking, and depends on others for feeding, toileting, and dressing. | 4, 5 | MoCA>=24 or MMSE>=26 | 0.552 (0.377–0.707) |
| **Long-term consequences, severe plus cognition problems** | Is confined to bed or a wheelchair, depends on others for feeding, toileting, and dressing, and has difficulty speaking, thinking clearly, and remembering things. | 4, 5 | MoCA<24 or MMSE<26 | 0.588 (0.411–0.744) |

MMSE: Mini-Mental State Examination; MoCA: Montreal Cognitive Assessment
