# Supplementary material for: The burden of stroke and its attributable risk factors in the Middle East and North Africa region, 1990–2019
Source: Sci Rep. 2022 Feb 17;12:2700. doi: 10.1038/s41598-022-06418-x (PMC8854638; doi:10.1038/s41598-022-06418-x)
Supplement: Supplementary file 11 — Supplementary Table S3. [file 41598_2022_6418_MOESM11_ESM.pdf]

Table S3: Prevalence of stroke in 1990 and 2019 for both sexes and the percentage change in the age-standardised rates (ASRs) per 100,000 in the North Africa and the Middle East region (Generated from data available from http://ghdx.healthdata.org/gbd-results-tool).					
	1990		2019		Percentage change in ASRs per 100,000
	No (95% UI)	ASRs per 100,000 (95% UI)	No (95% UI)	ASRs per 100,000 (95% UI)	
Global	54747917 (50221314 , 59562109)	1320.8 (1212.4 , 1440.5)	101474558 (93211910 , 110526302)	1240.3 (1139.7 , 1353)	-6.1 (-7.2 , -5)
North Africa and Middle East	3024732 (2813282 , 3261908)	1545.9 (1434.3 , 1673.3)	7323421 (6794727 , 7863138)	1537.5 (1421.9 , 1659.9)	-0.5 (-2.3 , 1.1)
Afghanistan	122216 (113555 , 131443)	1597.6 (1485.6 , 1723.9)	281177 (260881 , 302740)	1657.6 (1533.4 , 1779.5)	3.8 (-1.2 , 8)
Algeria	240104 (222748 , 257560)	1724.6 (1603.2 , 1858.9)	549736 (506627 , 592960)	1540.3 (1417.2 , 1668)	-10.7 (-14.5 , -7)
Bahrain	3704 (3414 , 4006)	1499.7 (1387.3 , 1636.7)	14539 (13369 , 15844)	1136.4 (1049 , 1234.8)	-24.2 (-27.5 , -20.8)
Egypt	504367 (467855 , 545560)	1523.8 (1408.7 , 1660.1)	1269834 (1170811 , 1374639)	1806.1 (1658.1 , 1974.5)	18.5 (12.9 , 23.9)
Iran (Islamic Republic of)	431372 (383019 , 483606)	1446.9 (1279.8 , 1649.5)	963512 (859232 , 1079662)	1253.8 (1113.5 , 1418.4)	-13.3 (-15.9 , -10.7)
Iraq	192900 (179987 , 206361)	2177.9 (2027.8 , 2349.2)	520023 (483072 , 557268)	1968.8 (1823.4 , 2122.1)	-9.6 (-13.3 , -5.4)
Jordan	37547 (34844 , 40187)	2342.5 (2150.9 , 2519.7)	134580 (123260 , 145467)	1793.9 (1621.2 , 1952.9)	-23.4 (-27.7 , -19.5)
Kuwait	11625 (10711 , 12622)	1307.7 (1215 , 1402.4)	42739 (39334 , 46356)	1230.6 (1134.6 , 1332.5)	-5.9 (-10.4 , -1.4)
Lebanon	33382 (30919 , 35892)	1395 (1289.8 , 1503.7)	74841 (69385 , 80771)	1425.1 (1320.3 , 1538.1)	2.2 (-1.8 , 6.6)
Libya	29993 (27884 , 32381)	1393.6 (1294.4 , 1505.9)	90351 (83440 , 96922)	1588.4 (1461.7 , 1712.9)	14 (8.9 , 18.7)
Morocco	252946 (235352 , 272302)	1638.9 (1517 , 1774.3)	557119 (515485 , 599727)	1695.1 (1563.4 , 1833.2)	3.4 (-0.7 , 8.2)
Oman	14093 (13024 , 15374)	1666.8 (1535.5 , 1837.5)	37722 (34606 , 41220)	1525.6 (1399.9 , 1686.6)	-8.5 (-12.4 , -4.5)
Palestine	15462 (14213 , 16720)	1569.1 (1439.1 , 1706)	41305 (37846 , 45067)	1510.8 (1381.5 , 1666.5)	-3.7 (-8.5 , 1.4)
Qatar	3339 (3061 , 3630)	1575.7 (1457.5 , 1705.2)	22335 (20296 , 24525)	1226.3 (1127.4 , 1327.2)	-22.2 (-25.6 , -18.9)

**Saudi Arabia**	**141842<br>(127066 , 160582)**	**1823.4<br>(1631.5 , 2105.7)**	**480500<br>(442193 , 523176)**	**1967.7<br>(1818.1 , 2143.5)**	**7.9<br>(-0.1 , 15.2)**
**Sudan**	**177207<br>(164323 , 190896)**	**1650.5<br>(1527.1 , 1787.5)**	**398038<br>(367084 , 427930)**	**1785.8<br>(1645.2 , 1936.9)**	**8.2<br>(3.7 , 12.8)**
**Syrian Arab Republic**	**111550<br>(103635 , 119384)**	**1763.3<br>(1639.3 , 1893.7)**	**199599<br>(184381 , 215112)**	**1518.7<br>(1403.8 , 1637.5)**	**-13.9<br>(-17.3 , -10.5)**
**Tunisia**	**56128<br>(51155 , 60995)**	**1032.7<br>(941.1 , 1123.3)**	**159928<br>(147442 , 173396)**	**1260.8<br>(1162.6 , 1366)**	**22.1<br>(16.3 , 28.2)**
**Turkey**	**531336<br>(492582 , 571402)**	**1328.5<br>(1232.1 , 1434.1)**	**1080379<br>(1002616 , 1164932)**	**1213.6<br>(1124.7 , 1309.4)**	**-8.7<br>(-12.4 , -4.6)**
**United Arab Emirates**	**16652<br>(15355 , 18115)**	**2405.6<br>(2215 , 2643.8)**	**136808<br>(125534 , 148340)**	**2225.7<br>(2037.3 , 2436.3)**	**-7.5<br>(-11.6 , -3.6)**
**Yemen**	**94933<br>(88038 , 102136)**	**1596.2<br>(1482.1 , 1716.9)**	**260915<br>(242029 , 280821)**	**1609.4<br>(1480.2 , 1745.5)**	**0.8<br>(-4.2 , 5.7)**
