# Supplementary material for: The burden of stroke and its attributable risk factors in the Middle East and North Africa region, 1990–2019
Source: Sci Rep. 2022 Feb 17;12:2700. doi: 10.1038/s41598-022-06418-x (PMC8854638; doi:10.1038/s41598-022-06418-x)
Supplement: Supplementary file 12 — Supplementary Table S4. [file 41598_2022_6418_MOESM12_ESM.pdf]

Table S4: Deaths from stroke in 1990 and 2019 for both sexes and the percentage change in the age-standardised rates (ASRs) per 100,000 in the North Africa and the Middle East region (Generated from data available from http://ghdx.healthdata.org/gbd-results-tool).					
	1990		2019		Percentage change in ASRs per 100,000
	No (95% UI)	ASRs per 100,000 (95% UI)	No (95% UI)	ASRs per 100,000 (95% UI)	
Global	4574085 (4295926 , 4892808)	132.4 (123.1 , 141.7)	6552725 (5995200 , 7015139)	84.2 (76.8 , 90.2)	-36.4 (-41.6 , -31.2)
North Africa and Middle East	177871 (159935 , 195301)	121.4 (105.8 , 134.5)	312220 (278450 , 349726)	87.7 (78.2 , 97.6)	-27.8 (-35.4 , -16)
Afghanistan	12072 (8312 , 15669)	198.6 (135.7 , 257.5)	16816 (11747 , 21934)	161.5 (110.3 , 208.5)	-18.7 (-36.1 , -1.6)
Algeria	14008 (11454 , 17145)	179.4 (149.3 , 211.2)	24811 (19995 , 30219)	101.5 (82.6 , 121.6)	-43.4 (-54.9 , -30.2)
Bahrain	117 (102 , 134)	102.5 (90.2 , 118.9)	245 (200 , 310)	52.8 (43.5 , 66.9)	-48.5 (-58.4 , -36.5)
Egypt	34626 (29818 , 39550)	110.8 (93.9 , 129)	45767 (33557 , 63157)	85.7 (63.1 , 118.9)	-22.6 (-42.6 , 1.6)
Iran (Islamic Republic of)	21698 (18964 , 23908)	120.7 (102.8 , 133.5)	40912 (36741 , 43849)	66.2 (58.7 , 71.3)	-45.1 (-50.6 , -35.4)
Iraq	11731 (9897 , 13689)	166.1 (140.6 , 193.9)	26256 (21422 , 31075)	143.3 (119.2 , 166.1)	-13.7 (-30.2 , 4.5)
Jordan	1426 (1185 , 1662)	150.9 (123.6 , 175.4)	3367 (2758 , 3983)	75.7 (61.3 , 89)	-49.8 (-59.4 , -39.2)
Kuwait	229 (202 , 252)	51.5 (44.4 , 57.6)	920 (757 , 1093)	46.5 (38 , 55.2)	-9.6 (-24.2 , 7.7)
Lebanon	974 (820 , 1163)	58.1 (48.4 , 69.3)	1764 (1236 , 2298)	35.2 (24.5 , 45.6)	-39.5 (-55.5 , -19.2)
Libya	1565 (1250 , 1936)	84.6 (66.2 , 107)	3086 (2364 , 4016)	69.4 (53 , 90.1)	-17.9 (-36.4 , 7.9)
Morocco	14768 (11718 , 17757)	132.2 (102.8 , 158.4)	29033 (23331 , 35135)	116.4 (94.2 , 139.3)	-11.9 (-29.1 , 8)
Oman	754 (581 , 955)	145.7 (112.8 , 183)	1030 (895 , 1196)	103.7 (90.7 , 118.7)	-28.8 (-45 , -3.7)
Palestine	1264 (1026 , 1532)	172.5 (141 , 208.3)	2019 (1740 , 2286)	122.4 (105.6 , 138.2)	-29.1 (-43.5 , -10.1)
Qatar	61 (48 , 79)	83.5 (63.4 , 108.7)	181 (136 , 239)	52.9 (42.5 , 69.2)	-36.7 (-54 , -15.7)

**Saudi Arabia**	**6915 (5388 , 8557)**	**152.2 (119.3 , 186.2)**	**12669 (9750 , 15359)**	**102.7 (80.4 , 120.5)**	**-32.5 (-49.1 , -10)**
**Sudan**	**14579 (11025 , 18829)**	**177.4 (127.6 , 228.6)**	**19638 (14425 , 27765)**	**125.2 (92.6 , 174.9)**	**-29.4 (-42.2 , -12)**
**Syrian Arab Republic**	**7385 (6165 , 8746)**	**146 (120.2 , 176.6)**	**9186 (7117 , 11838)**	**99 (78.3 , 124.9)**	**-32.2 (-50.2 , -8.6)**
**Tunisia**	**4063 (3398 , 4805)**	**106.6 (88.9 , 126.5)**	**8713 (6627 , 11147)**	**80 (60.6 , 101.4)**	**-25 (-43.8 , -0.6)**
**Turkey**	**22143 (18070 , 29102)**	**72.6 (58.2 , 96.1)**	**48947 (39204 , 59511)**	**60.6 (48.7 , 73.6)**	**-16.5 (-39.5 , 6.2)**
**United Arab Emirates**	**483 (368 , 678)**	**182.5 (145.5 , 246.2)**	**2168 (1544 , 3026)**	**91.3 (70.2 , 118.7)**	**-50 (-62 , -34.9)**
**Yemen**	**6892 (5025 , 8969)**	**174.2 (121.5 , 227.9)**	**14375 (10909 , 18865)**	**135.7 (102.4 , 176.6)**	**-22.1 (-38.2 , 0.7)**
