# Supplementary material for: The burden of stroke and its attributable risk factors in the Middle East and North Africa region, 1990–2019
Source: Sci Rep. 2022 Feb 17;12:2700. doi: 10.1038/s41598-022-06418-x (PMC8854638; doi:10.1038/s41598-022-06418-x)
Supplement: Supplementary file 13 — Supplementary Table S5. [file 41598_2022_6418_MOESM13_ESM.pdf]

Table S5: DALYs due to stroke in 1990 and 2019 for both sexes and the percentage change in the age-standardised rates (ASRs) per 100,000 in the North Africa and the Middle East region. DALY= disability-adjusted-life-years. (Generated from data available from http://ghdx.healthdata.org/gbd-results-tool).					
	1990		2019		Percentage change in ASRs per 100,000
	No (95% UI)	ASRs per 100,000 (95% UI)	No (95% UI)	ASRs per 100,000 (95% UI)	
Global	108206654 (102415590 , 114799148)	2729.9 (2579.9 , 2901.2)	143232184 (133095809 , 153241824)	1768.1 (1640.6 , 1889.4)	-35.2 (-40.5 , -30.5)
North Africa and Middle East	5543905 (5046736 , 6029506)	2685.3 (2433.7 , 2947.3)	7946004 (7060209 , 8870766)	1826.2 (1635.3 , 2026.2)	-32 (-39.1 , -23.3)
Afghanistan	324968 (236526 , 415319)	4471.6 (3212.1 , 5710.6)	507837 (375947 , 659772)	3498.2 (2508.8 , 4500.4)	-21.8 (-39.4 , -3.6)
Algeria	370111 (305580 , 443675)	3167.5 (2642 , 3755.9)	543945 (450965 , 649230)	1755.4 (1459.6 , 2081.9)	-44.6 (-55 , -31.9)
Bahrain	3785 (3376 , 4272)	1981.3 (1768.7 , 2241.6)	7987 (6663 , 9690)	940.9 (796.8 , 1146.2)	-52.5 (-61.2 , -42.5)
Egypt	1613654 (1260360 , 1974374)	3316 (2889.1 , 3785.7)	1493970 (1146402 , 1946512)	2138 (1636.8 , 2809.8)	-35.5 (-50 , -17)
Iran (Islamic Republic of)	597142 (547356 , 651017)	2324.3 (2051.8 , 2547)	884768 (812248 , 943655)	1262.2 (1153.5 , 1346.3)	-45.7 (-51 , -38.3)
Iraq	313596 (268605 , 361460)	3625.9 (3103.7 , 4212.9)	682943 (548026 , 826273)	2922.9 (2399 , 3459.1)	-19.4 (-35.5 , -0.8)
Jordan	36836 (31690 , 42514)	2910 (2466.9 , 3357.8)	86118 (72852 , 100150)	1448 (1220.4 , 1679.5)	-50.2 (-58.7 , -41.1)
Kuwait	7651 (6893 , 8401)	1062.6 (953.7 , 1169.4)	25463 (21787 , 29824)	938.4 (795 , 1093.3)	-11.7 (-24 , 3.3)
Lebanon	24063 (20791 , 28154)	1135.7 (980.3 , 1327.2)	39216 (30874 , 48709)	752.9 (593.3 , 935.9)	-33.7 (-47.6 , -16)
Libya	53989 (43012 , 67225)	1996.7 (1628.5 , 2421.5)	82320 (64181 , 104686)	1570.5 (1237.3 , 2001.2)	-21.3 (-37.5 , 1.4)
Morocco	387163 (322955 , 461398)	2727.6 (2237.6 , 3235.4)	666284 (534574 , 808810)	2257.8 (1838.8 , 2702.5)	-17.2 (-33.9 , 0.9)
Oman	24370 (19405 , 30109)	3071.8 (2428.5 , 3824)	30859 (27010 , 36651)	1884.3 (1667.3 , 2127.1)	-38.7 (-51.9 , -20.7)
Palestine	27471 (22492 , 33079)	3154.1 (2576 , 3790.5)	44902 (39457 , 50763)	2128.3 (1878.2 , 2399.7)	-32.5 (-46.4 , -15)
Qatar	2523 (2095 , 3130)	1635.2 (1336.8 , 2028.2)	8998 (7254 , 11097)	904.5 (747.6 , 1130.3)	-44.7 (-57.5 , -29.5)

**Saudi Arabia**	**182379 (143752 , 225284)**	**2982.3 (2362.6 , 3635)**	**417599 (326188 , 509857)**	**2114.9 (1685.1 , 2485.5)**	**-29.1 (-46.4 , -5.6)**
**Sudan**	**429900 (336046 , 549190)**	**3858.9 (2972.6 , 4948.3)**	**522412 (385879 , 715571)**	**2585.5 (1970.5 , 3552.9)**	**-33 (-45.3 , -16.9)**
**Syrian Arab Republic**	**252719 (210208 , 297392)**	**3375.3 (2823.7 , 3954.9)**	**238333 (184920 , 304410)**	**2018.1 (1589.6 , 2547.1)**	**-40.2 (-55.3 , -19.9)**
**Tunisia**	**96577 (82234 , 112798)**	**1961.4 (1663.1 , 2298.3)**	**176891 (135395 , 225499)**	**1477.5 (1138.5 , 1875)**	**-24.7 (-42.8 , -2)**
**Turkey**	**566638 (488799 , 711293)**	**1520.5 (1299.1 , 1911)**	**993082 (820881 , 1177528)**	**1162.6 (965 , 1380.4)**	**-23.5 (-42.5 , -5.2)**
**United Arab Emirates**	**18494 (14520 , 24703)**	**3565.6 (2877.6 , 4746.3)**	**95518 (70890 , 128945)**	**1925.6 (1517 , 2457.9)**	**-46 (-58.5 , -30.5)**
**Yemen**	**206145 (157464 , 261474)**	**3663.8 (2666.7 , 4746.9)**	**388486 (302567 , 500074)**	**2765.5 (2161.6 , 3520.5)**	**-24.5 (-41 , -1.9)**
